# Isotopic Characterization of 100% Agave Tequila (Silver, Aged and Extra-Aged Class) for Its Use as an Additional Parameter in the Determination of the Authenticity of the Beverage Maturation Time

**DOI:** 10.3390/molecules26061719

**Published:** 2021-03-19

**Authors:** Rocío Fonseca-Aguiñaga, Walter M. Warren-Vega, Floriberto Miguel-Cruz, Luis A. Romero-Cano

**Affiliations:** 1 Grupo de Investigación en Materiales y Fenómenos de Superficie, Departamento de Biotecnológicas y Ambientales, Universidad Autónoma de Guadalajara, Av. Patria 1201, Zapopan, Jalisco C.P. 45129, Mexico; rfonseca@crt.org.mx (R.F.-A.); wwarrenv12@gmail.com (W.M.W.-V.); 2 Laboratorio de Isotopía, Consejo Regulador del Tequila A. C. Av. Patria 723, Zapopan, Jalisco C.P. 45030, Mexico; fmiguel@crt.org.mx

**Keywords:** 100% agave tequila, authentication, maturation time, isotopic ratio, Jalisco regions

## Abstract

Isotopic ratios of δ^13^C_VPDB_ and δ^18^O_VSMOW_ have been used as an additional parameter to ensure the authenticity of the aging time of 100% agave tequila. For this purpose, 120 samples were isotopically analyzed (40 silver class, 40 aged class, and 40 extra-aged classes). The samples were obtained through a stratified sampling by proportional allocation, considering tequila producers from the main different regions of Jalisco, Mexico (Valles 41%, Altos Sur 31%, Cienega 16%, and Centro 12%). The results showed that the δ^13^C_VPDB_ was found in an average of −12.85 ‰ for all the analyzed beverages, with no significant difference between them. Since for all the tested samples the *Agave tequilana* Weber blue variety was used as source of sugar to obtain alcohol, those results were foreseeable, and confirm the origin of the sugar source. Instead, the results for δ^18^O_VSMOW_ showed a positive slope linear trend for the aging time (silver class 19.52‰, aged class 20.54‰, extra-aged class 21.45‰), which is associated with the maturation process, there are oxidation reactions that add congeneric compounds to the beverage, these can be used as tracers for the authenticity of the aging time. Additionally, the experimental data showed homogeneity in the beverages regardless of the production region, evidencing the tequila industry’s high-quality standards. However, a particular case occurs with the δ^18^O_VSMOW_ data for the silver class samples, in which a clear trend is noted with the altitude of the region of origin; therefore, this information suggests that this analytical parameter could be useful to authenticate the regional origin of beverage.

## 1. Introduction

Tequila has gained a high position in the international markets, being a high-quality beverage, achieved and maintained thanks to current legislation. According to these regulations [1], there are two categories of tequila: (a) 100% agave tequila, which uses 100% sugars from the *Agave tequilana* Weber blue variety, and (b) tequila, which corresponds to an alcoholic beverage in which 51% of its sugars are from the *Agave tequilana* Weber blue variety, and 49% comes from another source. Likewise, according to the maturation process’s characteristics, five classes can be defined: (a) *“Blanco”* (Silver): Transparent product but not necessarily colorless, without additives, obtained through distillation, whose commercial alcohol content must be adjusted by dilution with water. (b) *“Joven u Oro”* (Gold): Product that results from blending silver tequila with additives permitted by the official standard or from the mix of silver tequila with aged and/or extra-aged and/or ultra-aged tequilas. (c) *“Reposado”* (aged): A product which may be enhanced by mellowing, subject to an aging process of at least two months in direct contact with the wood from oak or Encino casks. (d) *“Añejo”* (extra-aged): Product which may be enhanced by mellowing in an aging process for at least one year in wood or oak recipients with V ≤ 600 L. (e) *“Extra añejo”* (ultra-aged): Product which may be enhanced by mellowing in an aging process for at least three years in wood or oak recipients with V ≤ 600 L. The production process is continuously regulated and verified by the *Consejo Regulador del Tequila A.C.* (Tequila Regulatory Council) to guarantee the consumer the authenticity and quality of the beverage, its raw materials, and the legal requirements that protect the appellation of origin. In recent years the CRT has documented counterfeit tequila cases in different parts of the world, which has led to the destruction of these products in the United States of America, Uruguay, Greece, France, Germany and Chile [2]. The economic problems caused to the industry formally established by the illegal sale of fraudulent beverages are evident and estimated in millions of pesos; however, the damage caused to the tequila brand is even more relevant since it directly affects the image of the agave tequila sector internationally, and potentially puts the consumer at risk.

To solve the problems of adulteration and counterfeiting of the beverage, numerous research groups have focused their efforts to propose analytical techniques such as profiles of volatile organic compounds [3], gas chromatography-olfactometry [4], analysis by near-infrared spectroscopy (NIR-SIMCA) [5], solid-phase micro extraction- chromatography-mass spectrometry (SPME-GC-MS) [6], stable isotope evaluation [7], fractionation of natural isotopes of the specific site studied by nuclear magnetic resonance (SNIF-NMR) [8], solid-phase microextraction (SPME) [9,10,11], infrared spectroscopy by Fourier transform (FTIR) [12,13], chemometric studies and spectroscopic techniques [14], studies using photo-acoustic [15], studies using surface plasmon resonance [16], fluorescence spectroscopy [17], and through pattern recognition and supervised classification [18]. That can possibly use as an additional criterion for the 100% agave tequila silver class’s authenticity. The next step is to obtain analytical parameters that may be useful to determine the authenticity of aged and extra-aged class 100% agave tequila. Therefore, some research groups have proposed the use of some organic compounds, like higher alcohols and esters, as authenticity markers since their concentration in the beverage increases or decreases due to aging time [18,19,20,21,22,23]. However, the techniques mentioned above are not reliable because, in most non-authentic beverages, the chemical manipulation of ethanol has been detected through the addition of organic components, making these fake beverages pass as legitimate since they achieve compliance with the physicochemical parameters established by current regulations. For this reason, in the present study, the use of gas chromatography—isotope ratio mass spectrometry is presented as a strategy to obtain more sensitive chemical tracers that may be useful to authenticate the maturation time of 100% agave tequila. For this purpose, 120 samples of 100% agave tequilas from different regions of the state of Jalisco (Mexico) were studied.

## 2. Results and Discussion

### 2.1. Study of Different Classes of Tequila (Silver, Aged, and Extra-Aged)

**Figure 1:**
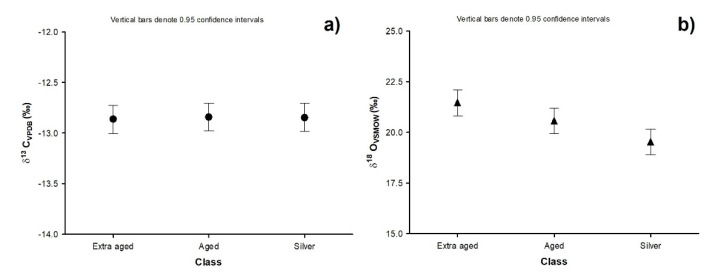
Statistical analysis of the values δ^13^C_VPDB_ (**a**) δ^18^O_VSMOW_ (**b**), different classes of 100% agave tequila (silver, aged, and extra-aged).

Graphical representation of the experimental data for δ^13^C_VPDB_ and δ^18^O_VSMOW_, according to the year of production of silver, aged, and extra-aged 100% agave tequila are presented in Figure S1. It is appreciated that there is no statistically significant difference between the years of production. From the information presented in the Figure, the statistical analyzes presented in the Figure 1 and Table 1 were performed. It is observed that the values are in the order of −12.84 ± 0.54‰ for silver class, −12.84 ± 0.41‰ for aged class, and −12.86 ± 0.34‰ for extra-aged class (Figure 1a). When comparing the means of all the samples, no significant differences were detected (*p* = 0.96 > 0.05), which corroborates the authenticity of the three classes of tequila analyzed since the values obtained are in the range previously reported [2]; from these results, it is possible to determine the type of plant used as a sugar source during the fermentation step. According to different authors [2,24,25,26] depending on the photosynthetic process to fix atmospheric CO_2_, plants are classified into three groups: (i) C3 plants that use the Calvin cycle, whose values of δ^13^C have been reported in a range of −22 to −33‰; (ii) C4 plants, that follow the Hatch Slack cycle, whose values of δ^13^C are within a range of −10 to −20‰; (iii) Crassulacean Acid Metabolism (CAM) plants which have a δ^13^C in a range of −12 to −30‰, of this group, the species that fix CO_2_ at night stand out, which have a carbon delta close to 13‰ very similar to the experimental results obtained in the range between −12.0 to 13.5‰, these values can be related to the species of *Agave tequilana* Weber blue variety used in the sugar fermentation process.

Due to the homogeneity of the experimental data, the δ^13^C_VPDB_ value can only be considered as an additional parameter that corroborates the sugar source’s authenticity used to make tequila. However, a particular phenomenon can be seen when analyzing the experimental data obtained for δ^18^O_VSMOW_ (Figure 1b and Table 1). The mean value obtained for the silver class samples was 19.52‰, which is related to the conversion of sugars from the *Agave tequilana* Weber blue variety to ethanol. The data show significant differences between the groups (*p* < 0.05) as well as a positive slope linear trend with the aging time: 19.52 ± 2.14 ‰ for silver class, 20.54 ± 1.89‰ for aged class, and 21.45 ± 2.03‰ for extra-aged class.

This isotopic enrichment can be explained due by the oxidation reactions that the beverage undergoes during the maturation process, similarly to the one proposed by Warren-Vega et al. [26]. Silver tequila is placed in oak casks wherein a first stage the ethanol is oxidized forming acetaldehyde (Reaction 1), which at low concentrations provides the characteristic fruity aroma, influencing the chemical profile of the beverage, triggering the oxidation of polyphenols such as anthocyanins and flavanols affecting color and aroma [27]. After that, the acetaldehyde is oxidized to form acetic acid (Reaction 2) which it combines with ethanol to form ethyl acetate (Reaction 3), which adds aroma to the beverage [28]. These reactions are present throughout the maturation process, which is because their concentration increases over time. In the pretreatment of samples, distillation is carried out at 78 °C; for this reason, the value reported is the combination of δ^18^O_VSMOW_ of the ethanol molecule and δ^18^O_VSMOW_ of the ethyl acetate molecule since both present the same boiling point. In this way, it is possible to explain the isotopic enrichment in determining tequila classes with different aging times.
(R1)
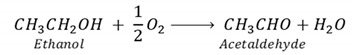

(R2)
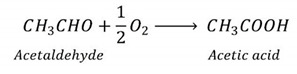

(R3)
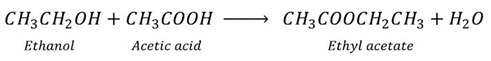



**Figure 2:**
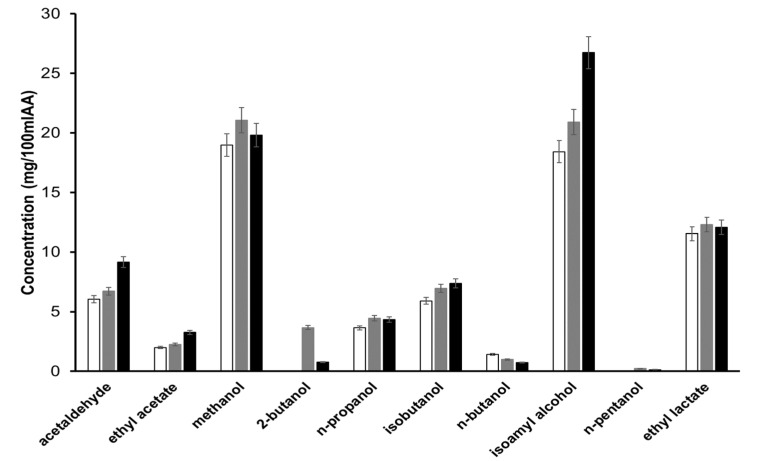
Chromatographic characterization of the 100% agave tequila congeners; class: □ silver, ■ aged, and ■ extra-aged.

The previous statements can be corroborated with the characterization of congeners of the studied beverages, Figure 2. It shows the evolution of acetaldehyde and ethyl acetate formation linearly with the beverage’s maturation time. Therefore, the combination of both analytical techniques (GC-FID and GC-IRMS) can be proposed as a complementary method to determine the tequila’s aging time’s authenticity.

### 2.2. Study of the Different Regions of Tequila Production

**Figure 3:**
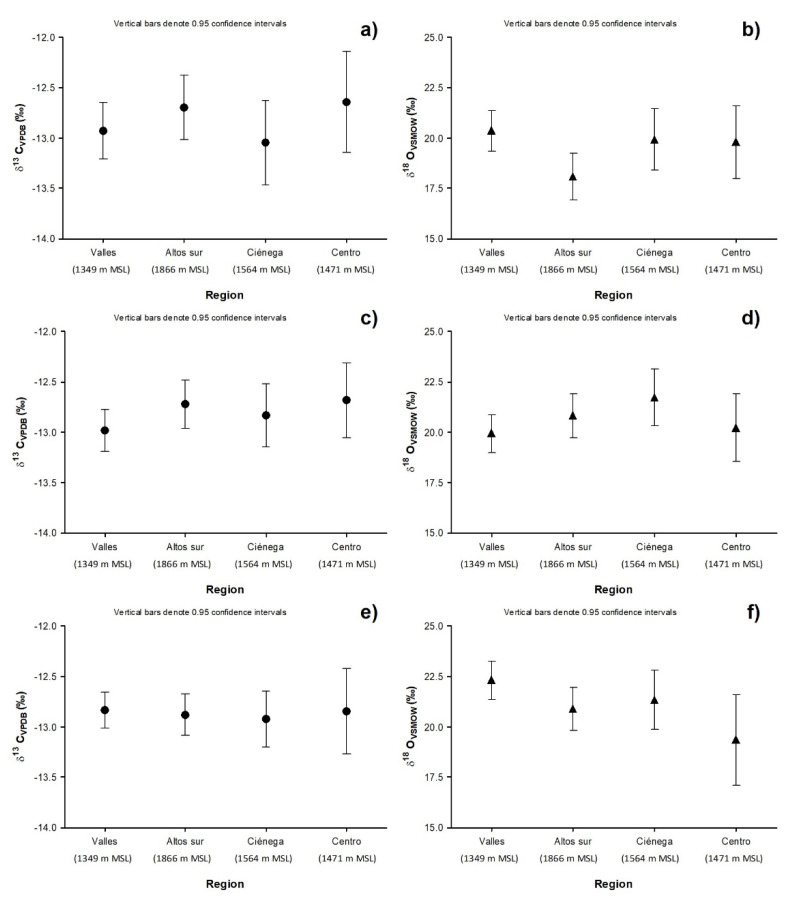
Statistical analysis of the values of δ^13^C_VPDB_ and δ^18^O_VSMOW_, for tequila 100% agave silver class (**a**,**b**), aged class (**c**,**d**), and extra-aged class (**e**,**f**).

To find authenticity tracers to distinguish the origin of the beverage, the experimental data were analyzed by comparing the mean value of each of the regions (Valles, Altos Sur, Cienega, and Centro) for each of the 100% agave tequila classes. Graphical representation of the experimental data for δ^13^C_VPDB_ and δ^18^O_VSMOW_, according to the year of production (2016–2019) of silver, aged, and extra-aged 100% agave tequila are presented in Figure S2. It is appreciated that there is no statistically significant difference between the years of production. From the information presented in the figure, the statistical analyzes presented in Figure 3 and Table 2 were performed.

In the case of δ^13^C_VPDB_, in every of the studied classes, no significant differences were observed (*p* > 0.05, in all cases), which suggests that all the studied companies, regardless of the region of origin, meet the additional criteria of authenticity of the carbon source used to produce the beverage, since all values can be related to the *Agave tequilana* Weber blue variety.

**Figure 4:**
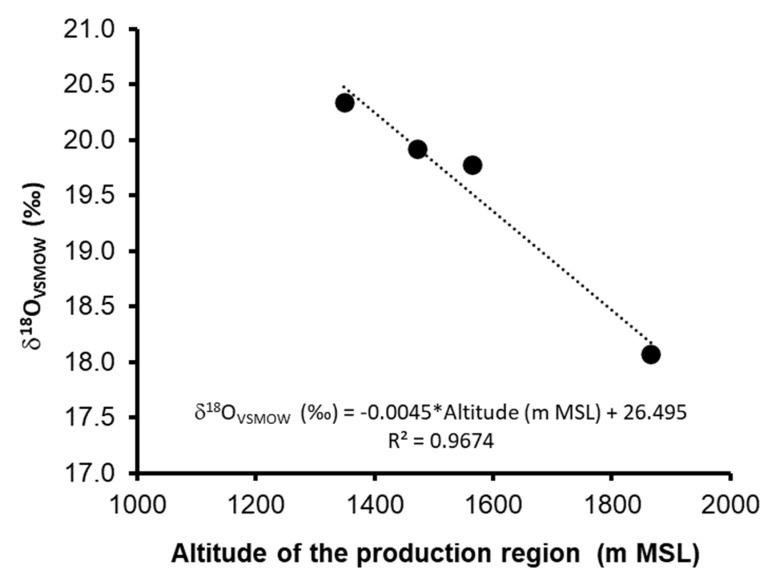
Relationship between the altitude of the production region of silver class 100% agave tequila and the δ^18^O_VSMOW._

A particular case occurs with the data for the silver class 100% agave tequila samples, which present significant differences between groups (*p* = 0.03 < 0.05). Performing the experimental data analysis, it is appreciated that there is linearity between the region’s average altitude [29] versus the average values of δ^18^O_VSMOW_ (Figure 4). This effect can be attributed to the last stages of the tequila production process: distillation. The distillation process is linked to the altitude of the place where it is carried out because it is related to temperature. As the pressure decreases, with the increase in altitude, the system requires a more significant decrease in temperature to be able to reach the saturated water vapor pressure [30] in such a way that the water molecules constituted by the light isotopes of O and H will stay mostly in the vapor phase concerning the liquid phase, for this reason, the δ^18^O_VSMOW_ of the distillate is enriched. Another possibility to which the phenomenon could be attributed is the water used in the process. To rule out this possibility, the δ^18^O_VSMOW_ of the water used in each of the regions was analyzed; however, the data obtained: Valles (1349 m MSL vs. −9.50 ‰), Centro (1471 m MSL vs. −9.36 ‰), Cienega (1564 m MSL vs. −9.07 ‰), Altos Sur (1866 m MSL vs. −9.49 ‰) show that there is no significant difference between regions, in such a way that the observed effect can be attributed mainly to the distillation process. It is important to note that this effect cannot be observed in the samples of aged and aged tequila because, at the time of aging in the barrel, the hydro-alcoholic matrix begins to become more complex due to incorporating oxidation reactions products previously discussed (Reaction 1 to 3).

Although it is necessary to characterize a more significant number of samples to be able to clearly define the averages and limits to identify the production region with more sensitivity, this research work presents the first studies in which the possibility of using the values of δ^18^O_VSMOW_ as an additional parameter to determine the authenticity of the region of origin of the white class 100% agave tequila.

## 3. Materials and Methods

### 3.1. Samples

**Figure 5:**
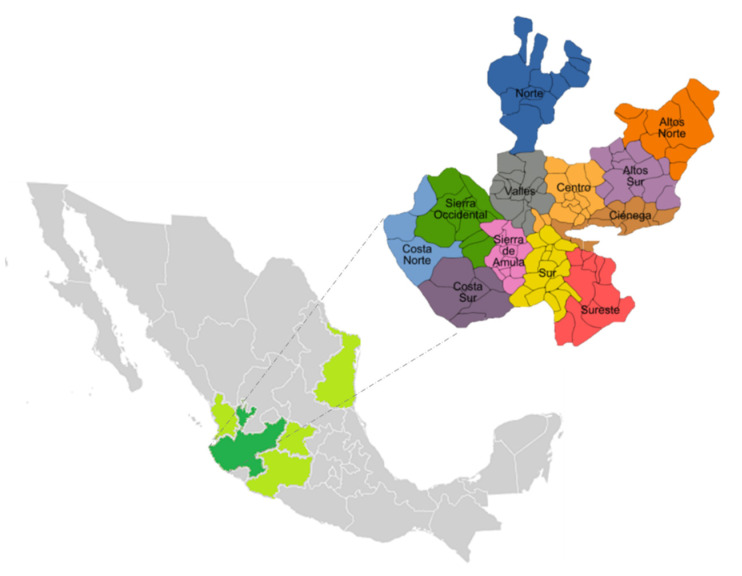
Map of Mexico states that conform to tequila’s appellation in green color: Nayarit (8 municipalities), Jalisco (125 municipalities), Michoacán (30 municipalities), Guanajuato (7 municipalities) and Tamaulipas (11 municipalities). Highlighting in the dark green the state of Jalisco with its division by geographical regions.

To obtain an analytical characterization of 100% agave tequila (silver, aged, and extra-aged class), stratified sampling was carried out by proportional allocation considering the distribution of tequila producers located in the appellation of origin area. For this purpose, the Consejo Regulador de Tequila A. C. (CRT, for its acronym in Spanish) requested random samples of the final product from different tequila producers (63 in total) located in the state of Jalisco (Mexico) during the years 2016 to 2019 (Table 3, Sup. Table 2, and Figure 5).

#### 3.1.1. Sample Preparation

To obtain ethanol at alcohol content greater than 92% m/m, the samples were distilled in the automatic distillation equipment using ADCS software before analyzing in the gas chromatograph. The ethanol obtained from the distillation was injected directly into the chromatograph equipment to determine the isotopic ratio of carbon 13 (δ^13^C_VPDB_). To determine oxygen 18 (δ^18^O_VSMOW_), it was necessary to contact the distilled sample in a molecular sieve for at least 6 h. Then, the sample was analyzed.

#### 3.1.2. Determination of the Isotopic Ratio by GC/IRMS

To determine the isotopic ratio of carbon (δ^13^C_VPDB_), the combustion reactor was programmed at a temperature of 960 °C. The conditions of the gas chromatograph were: helium flow of 2.3 mL min^−1^ at an initial temperature of 160 °C during 13 min, after that it was increased 10 °C min^−1^ until it reached a temperature of 250 °C and at the end, it was maintained for 27 min. The injection port temperature was 200 °C, and a 25:1 split. To determine the isotopic ratio of oxygen 18 (δ^18^O_VSMOW_), the high-temperature conversion reactor was programmed at a temperature of 1260 °C. The gas chromatograph conditions were: helium flow of 2.2 mL min^−1^ at an initial temperature of 160 °C for 9 min; after that, it was increased 12 °C min^−1^ reached a temperature of 250 °C, and it was maintained for 10.5 min. The injection port temperature was 200 °C and the split was 11:1. The auxiliary gas for pyrolysis was 2% helium hydrogen, with a flow of 0.20–0.25 mL min^−1^. All the list of reagents and gases for the systems utilized during the analytical determinations are presented in Supplementary Table S1. The obtained isotopic ratios were expressed in parts per mil (‰) by a normalization of the results obtained from the reference gas previously standardized concerning an international reference Vienna PD Belemnite (VPDB) and Vienna Standard Mean Ocean Water (VSMOW). The relative difference of isotope ratios (isotope-delta values) was obtained by Equation (1) [31]:

(1) δ(Ei/j)=δEi/j=Ri/jp−Ri/jrefRi/jref

where *^i^E* denotes the higher (superscript i) and *^j^E* the lower (superscript j) atomic mass number of element *E*. Isotope-delta values are small numbers and therefore frequently present in multiples of 10^−3^ or per mil (symbol ‰) [31]. According to the previous reference, the values for δ^13^C_VPDB_ and δ^18^O_VSMOW_ were obtained from Equations (2) and (3).

All values δ were expressed concerning the international reference Equations (1) and (2).

(2) δC13VPDB=[(C13/C12)sample(C13/C12)standard VPDB−1]

(3) δO18VSMOW=[(O18/O16)sample(O18/O16)standard VSMOW−1]

The methods used to determine the isotopic ratios of carbon 13 (δ^13^C_VPDB_) and oxygen 18 (δ^18^O_VSMOW_) were validated in the laboratory in accordance with the Eurachem guide “The Fitness for Purpose of Analytical Methods a Laboratory guide to Method Validation and Related Topics” [32]. Additionally, the laboratory approved an international inter calibration test (Profiency test Scheme 2017, with Eurofins sample code 17/2/E and Proficiency test Scheme 2019, with Eurofins sample code 19/2/E) which certifies its validation process. It is important to note that our laboratory maintains a quality control program where periodic reference materials and control samples are analyzed to ensure the quality of our results.

### 3.2. Chromatographic Analysis

The determination of aldehydes, methanol, esters, and higher alcohols was carried out by the chromatographic method shown by the standard Mexican NMX-V-005-NORMEX-2018 [33]. Briefly, gas chromatographic analyses were carried out using an Agilent 7890 gas chromatograph, (Agilent Technologies, Boston, MA, USA) equipped with a flame ionization detector. The column used was an Agilent J&W DB-WAX (60 m × 0.25 mm, 0.25 μm). The chromatographic conditions were as follows: 40 °C for 4 min, increased at 5 °C min^−1^ to 100 °C, then 10 °C min^−1^ to 220 °C, and maintained at this temperature until a 10 min run was completed. A sample volume of 1.0 μL was automatically injected using helium as carrier gas with a flow rate of 1.3 mL min^−1^, nitrogen as auxiliary gas 30 mL min^−1^, air 450 mL min^−1^, and Hydrogen 40 mL min^−1^. Injector and detector temperatures were set at 200 °C and 220 °C, respectively. Quantitative data were obtained by interpolating peak areas using calibration plots built from the analysis of solutions containing a known amount of the analytes mentioned above.

### 3.3. Statistic Analysis

The statistical analysis of the experimental data was carried out using the STATISTICA 10 software (StatSoft, Palo Alto, CA, USA) employing a one-way analysis of variance (ANOVA) to determine the existence of statistically significant differences between the means of the groups (Tequila classes and production regions) using a significance level of 95% (*p* < 0.05).

## 4. Conclusions

It is possible to use the isotopic ratio of oxygen 18 (δ^18^O_VSMOW_) as an additional parameter to determine the authenticity of the aging time of 100% agave tequila. The average values for each of the classes are silver class (without maturation) 19.52‰, aged class (at least two months of aging), 20.54‰, extra-aged class (at least one year of aging) 21.45‰. Likewise, a particular case occurs with the δ^18^O_VSMOW_ data for the silver class samples, in which a clear trend is noted with the altitude of the region of origin. Therefore, this information suggests that this analytical parameter could be useful to authenticate the regional origin of this type of beverage. Finally, such homogeneous values in the δ^13^C_VPDB_ values (average −12.85‰) suggest that the CRT verification processes have a favorable impact on ensuring the authenticity and quality of the final product in the tequila industry; the additional parameter shows that the beverages come from the *Agave Tequilana* Weber blue variety plant.

## Figures and Tables

**Table 1:** One-way analysis of variance for δ^13^C_VPDB_ and δ^18^O_VSMOW_ in different classes of 100% agave tequila (silver, aged, and extra-aged class). SS: Sum-of-squares, DF: Degrees of freedom, MS: Mean squares, F: F-value = MS_intercept_/MS_error_, P: *p*-value.

**δ^13^C_VPDB_**					
**Effect**	**SS**	**DF**	**MS**	**F**	**P**
Intercept	19,816.36	1	19,816.36	100,418.7	0.0000
Class	0.01	2	0.01	0.0	0.9674
Error	23.09	117	0.20		
**δ^18^O_VSMOW_**					
**Effect**	**SS**	**DF**	**MS**	**F**	**P**
Intercept	50,468.73	1	50,468.73	12,281.15	0.0000
Class	74.79	2	37.39	9.10	0.0002
Error	480.81	117	4.11		

**Table 2:** One-way analysis of variance for δ^13^C_VPDB_ and δ^18^O_VSMOW_ in different classes of tequila 100% agave (silver, aged, and extra-aged class) grouping the data by region. SS: Sum-of-squares, DF: Degrees of freedom, MS: Mean squares, F: F-value = MS_intercept_/MS_error_, P: *p*-value.

**Silver Class (δ^13^C_VPDB_)**					
**Effect**	**SS**	**DF**	**MS**	**F**	**P**
Intercept	5388.58	1	5388.58	17,852.46	0.0000
Region	0.863	3	0.288	0.95	0.4252
Error	10.866	36	0.302		
**Silver Class (δ^18^O_VSMOW_)**					
**Effect**	**SS**	**DF**	**MS**	**F**	**P**
Intercept	12,492.77	1	12,492.77	3166.75	0.0000
Region	37.60	3	12.53	3.177	0.0356
Error	142.02	36	3.94		
**Aged Class (δ^13^C_VPDB_)**					
**Effect**	**SS**	**DF**	**MS**	**F**	**P**
Intercept	5368.92	1	5368.92	32,296.68	0.0000
Region	0.616	3	0.205	1.23	0.3114
Error	5.985	36	0.166		
**Aged Class (δ^18^O_VSMOW_)**					
**Effect**	**SS**	**DF**	**MS**	**F**	**P**
Intercept	13,995.19	1	13,995.19	4102.25	0.0000
Region	17.33	3	5.78	1.693	0.1857
Error	122.82	36	3.41		
**Extra-Aged (δ^13^C_VPDB_)**					
**Effect**	**SS**	**DF**	**MS**	**F**	**P**
Intercept	4331.06	1	4331.065	33,066.47	0.0000
Region	0.0444	3	0.015	0.11	0.9532
Error	4.715	36	0.131		
**Extra-Aged (δ^18^O_VSMOW_)**					
**Effect**	**SS**	**DF**	**MS**	**F**	**P**
Intercept	11,498.78	1	11,498.78	3154.77	0.0000
Region	29.82	3	9.94	2.727	0.0582
Error	131.22	36	3.64		

**Table 3:** Determination of the number of samples for tequila 100% agave (silver, aged, and extra-aged class) based on a stratified sampling method by proportional allocation taking into consideration the distribution of tequila producers located in Jalisco, Mexico.

Jalisco Region	Total Number of Tequila Producers Active in the Region	%	Samples (Silver Class)	%	Samples (Aged Class)	%	Samples (Extra Aged Class)	%
Valles	55	41	16	40	16	40	17	42
Altos sur	41	31	12	31	12	31	13	32
Ciénega	21	16	7	17	7	17	7	17
Centro	16	12	5	12	5	12	3	9
Total	133	100	40	100	40	100	40	100

## Data Availability

The data that support the findings of this study are available from the corresponding author upon reasonable request.

## Supplementary Materials

The following are available online. List of reagents and gases for the systems utilized during the analytical determinations; Supplementary Table S1. Operating conditions of the GC/C/IRMS and GC/HTC/IRMS analyzes, Figure Sup. 1. Relationship between the altitude of the production region of tequila 100% agave silver class and the δ^18^O_VSMOW_. Reference [34] is cited in the supplementary materials.

## Sample Availability

The samples that support the findings of this study are available from the corresponding author upon reasonable request.
