# Supplementary material for: Isotopic Characterization of 100% Agave Tequila (Silver, Aged and Extra-Aged Class) for Its Use as an Additional Parameter in the Determination of the Authenticity of the Beverage Maturation Time
Source: Molecules. 2021 Mar 19;26(6):1719. doi: 10.3390/molecules26061719 (PMC8003353; doi:10.3390/molecules26061719)
Supplement: Supplementary file 1 [file molecules-26-01719-s001.pdf]

molecules

MDPI

Article

Isotopic Characterization of Tequila 100% Agave (Silver, Aged and Extra-Aged Class) for its Use as an Additional Parameter in the Determination of the Authenticity of the Beverage Maturation Time

Rocío Fonseca-Aguiñaga[1,2], Walter M. Warren-Vega[1], Floriberto Miguel-Cruz[2] and Luis A. Romero-Cano[1,*]

[1] Grupo de Investigación en Materiales y Fenómenos de Superficie. Facultad de Ciencias Químicas. Universidad Autónoma de Guadalajara. Av. Patria 1201, C.P. 45129. Zapopan, Jalisco. MEXICO.
[2] Laboratorio de Isotopía. Consejo Regulador del Tequila A. C. Av. Patria 723, C.P. 45030. Zapopan, Jalisco. MEXICO

Supplementary Information

List of reagents and gases for the systems utilized during the analytical determinations

Reagents

- Secondary ethanol standard, traceable to the IAEA identified as BCR-656 with a value of $\delta^{13}C_{VPDB}$ of -26.91 ‰.
- Secondary standard of ethanol (from cane origin) identified as C2 with a value of ◎$^{13}C_{VPDB}$ of -12.83 ‰ and a value of $\delta^{18}O_{VSMOW}$ of 16.7 ‰.
- Secondary standard of absolute ethanol (Sigma Aldrich) identified as Absolute with a value of $\delta^{13}C_{VPDB}$ of -25.21 ‰ and a value of ◎$^{18}O_{VSMOW}$ of 17.7 ‰.
- Secondary standard of ethanol (from grape origin) identified as Grape with a value of $\delta^{13}C_{VPDB}$ of -27.21 ‰ and a value of $\delta^{18}O_{VSMOW}$ of 25.50 ‰.
- Secondary ethanol standards of different origins, obtained from the interlaboratory test identified as: Standard-1 with a value of $\delta^{18}O_{VSMOW}$ of 36.85 ‰, Standard-2 with a value of $\delta^{18}O_{VSMOW}$ of 18.50 ‰ and a Standard-3 with a $\delta^{18}O_{VSMOW}$ value of 27.05 ‰ [33,34]

- Standard prepared in the CRT laboratory known as Control Sample (tequila 100% Agave) with a value of $\delta^{13}C_{VPDB}$ of -13.01 ‰ and a value of $\delta^{18}O_{VSMOW}$ of 22.18 ‰.
- Standard from the proficiency test identified as 19/E with a value of $\delta^{18}O_{VSMOW}$ of 23.59 ‰ and $\delta^{13}C_{VPDB}$ of 23.59 ‰.

Gases for the systems

- Helium (99.999% purity), used as a carrier gas in the chromatograph and in the Isolink GC; as dilution gas in Conflo IV.
- Carbon dioxide (99.998% purity), used as reference or working gas during the On-Off Standard exercises.
- Oxygen (99.999% purity), used as a regenerating gas of the combustion reactor.
- Carbon dioxide with a known value of $\delta^{13}C$, to normalize the carbon dioxide tank with 99.998% purity, used as a working gas (or reference).
- Carbon monoxide (99.99% purity), used as reference or working gas during the On-Off Standard analysis.
- Mixture of: Hydrogen on 2% Helium (calibration standard grade), which meets the specifications of impurities such as helium gas (grade 5.0), used as an auxiliary gas in the thermo decomposition reaction.

Table 1. Operating conditions of the GC / C / IRMS and GC / HTC / IRMS analyzes.

Instrumentation	($\delta^{13}C$) GC/C/IRMS	($\delta^{18}O$) GC/HTC/IRMS
Automatic distiller equipment	Eurofins, with software ADCS	
Gas chromatograph	Thermo, Model: Trace 1310 with capillary injection system	
Autosampler	Thermo, Model: Triplus RSH	
Syringe	PAL System, Volume: 1.0 µl, PAL3-SyS-000680	

IRMS	Thermo, Model: Delta V Plus with triple Faraday collector for the simultaneous assignment of masses 44, 45 and 46 corresponding to CO_2 and masses 28, 29 and 30, corresponding to CO		
Interfaces	Thermo Scientific, model GC Isolink II. Equipped with an Al_2O_3 reactor filled with nickel oxide, copper oxide and platinum maintaining temperatures of 960 °C to carry out the combustion reaction. Also, with an Al_2O_3 reactor filled with nickel and platinum keeping at a temperature close to 1 260 °C to carry out the thermal decomposition reaction.<br>Thermo Scientific model Conflo IV, continuous flow coupled to the mass spectrometer of isotopic ratios.		
Mode	Continuous Flow, Configuration CO_2	Continuous Flow,	Configuration CO
Oven	Maximum temperature of 450 °C		
Column	PoraBond Q 50 m, from -100 ºC to 300 ªC, I.D. 0.32 mm, Film 5 µm. Open tubular column of porous layer with stationary phase PLOT, 50 m long, 0.45 mm external diameter, 0.32 mm internal diameter and 5 µm internal film		
**Chromatographic conditions**			
Carrier gas	Helium, constant flow, 2.3 mL min^{-1}	Helium, constant flow, 2.2 mL min^{-1}	
Temperature	160 °C during 13 min, 10 °C min^{-1} until 250 °C, total 48 min	160 °C during 9 min, 12 °C min^{-1} until 250 °C, total 25 min	
Injector	Mode: Split, Relation Split: 25:1, 200 °C.	Mode: Split, Relation Split: 11:1, 200 °C	
Other gases	Oxygen to regenerate the combustion reactor	Mixture of Hydrogen in He at 2% as auxiliary gas in the thermo decomposition reaction, 0.20-0.25 mL min^{-1}	
Reactor temperature	960 °C	1260 °C	
Injection volume	0.3 µl	1.0 µl	

Table 2. Compilation of the experimental data obtained in the present research work.

Tequila 100% agave class:	Year	Company identification	$\delta^{13}C_{VPDB}$ (‰)	$\delta^{18}O_{VSMOW}$ (‰)
Extra Aged	2016	48	-12.60	17.49
Extra Aged	2018	133	-12.82	19.14
Extra Aged	2018	133	-13.12	21.42
Extra Aged	2016	79	-13.10	17.23
Extra Aged	2016	117	-13.35	21.17
Extra Aged	2016	77	-12.19	21.04
Extra Aged	2016	101	-13.03	20.22
Extra Aged	2017	14	-12.91	19.14
Extra Aged	2017	28	-12.95	23.02
Extra Aged	2017	71	-12.97	20.52
Extra Aged	2018	12	-13.11	19.13
Extra Aged	2018	12	-13.01	21.73
Extra Aged	2018	12	-12.91	19.48
Extra Aged	2019	12	-12.81	22.16
Extra Aged	2019	12	-12.82	21.75
Extra Aged	2019	12	-12.28	24.96
Extra Aged	2016	39	-12.39	19.04
Extra Aged	2018	64	-13.33	21.65
Extra Aged	2018	64	-12.83	20.13
Extra Aged	2018	64	-13.23	22.97
Extra Aged	2018	64	-12.93	23.94
Extra Aged	2018	64	-12.53	21.60
Extra Aged	2018	64	-13.21	20.08
Extra Aged	2016	150	-13.29	18.84
Extra Aged	2016	94	-12.65	19.26
Extra Aged	2016	110	-12.49	21.72
Extra Aged	2016	137	-12.50	22.79
Extra Aged	2019	13	-12.75	23.63
Extra Aged	2019	126	-13.30	22.37
Extra Aged	2019	126	-12.70	24.64
Extra Aged	2019	126	-12.79	23.79
Extra Aged	2019	126	-12.74	21.75
Extra Aged	2019	13	-13.42	21.17
Extra Aged	2019	151	-13.33	24.15
Extra Aged	2018	43	-13.21	18.34
Extra Aged	2018	43	-12.94	23.93
Extra Aged	2018	13	-12.23	24.09
Extra Aged	2018	126	-13.03	22.65
Extra Aged	2018	126	-12.00	23.30
Extra Aged	2018	126	-12.81	22.73
Aged	2016	47	-12.19	21.04
Aged	2016	133	-13.03	17.76
Aged	2016	54	-12.28	21.42
Aged	2018	133	-13.47	20.95
Aged	2018	33	-12.45	19.92
Aged	2016	44	-13.10	17.23

Aged	2016	26	-12.49	21.72
Aged	2016	115	-12.50	22.79
Aged	2017	9	-12.91	19.48
Aged	2018	115	-13.23	22.97
Aged	2018	96	-12.93	23.94
Aged	2018	96	-12.67	23.97
Aged	2016	79	-13.35	18.49
Aged	2016	124	-12.87	19.86
Aged	2016	14	-13.35	21.17
Aged	2016	6	-12.60	17.49
Aged	2016	28	-12.59	20.99
Aged	2016	70	-12.72	21.51
Aged	2016	68	-12.97	20.52
Aged	2016	14	-13.03	22.65
Aged	2016	14	-12.00	23.30
Aged	2016	148	-12.81	22.73
Aged	2016	97	-12.31	20.01
Aged	2016	148	-12.09	21.16
Aged	2016	127	-13.29	18.84
Aged	2016	50	-12.65	19.26
Aged	2016	4	-12.23	23.04
Aged	2016	120	-12.39	19.04
Aged	2016	2	-13.03	20.22
Aged	2016	137	-12.95	23.02
Aged	2017	153	-13.12	21.42
Aged	2017	30	-12.83	20.13
Aged	2018	126	-12.53	21.60
Aged	2018	126	-13.21	20.08
Aged	2018	126	-13.49	19.42
Aged	2018	126	-12.99	17.82
Aged	2018	126	-13.04	17.44
Aged	2018	126	-13.41	17.43
Aged	2018	126	-13.55	19.91
Aged	2018	13	-13.02	20.22
Silver	2018	87	-12.85	15.85
Silver	2018	12	-11.34	16.44
Silver	2017	102	-12.94	16.43
Silver	2017	154	-13.53	16.48
Silver	2018	12	-11.73	18.00
Silver	2017	11	-13.35	17.03
Silver	2017	54	-11.37	18.70
Silver	2017	70	-12.34	19.02
Silver	2016	18	-13.26	17.44
Silver	2018	14	-13.55	20.86
Silver	2018	14	-13.06	20.93
Silver	2017	114	-13.05	19.68
Silver	2017	81	-12.36	16.22
Silver	2016	121	-13.32	18.62
Silver	2016	126	-13.27	18.75

Silver	2017	153	-12.91	20.64
Silver	2016	104	-12.57	19.67
Silver	2016	50	-13.27	19.86
Silver	2016	150	-13.10	19.89
Silver	2017	136	-12.62	21.31
Silver	2016	107	-12.94	20.20
Silver	2016	120	-12.76	20.33
Silver	2016	17	-12.80	20.39
Silver	2016	83	-13.42	20.71
Silver	2017	110	-12.72	22.36
Silver	2016	105	-13.10	21.86
Silver	2016	43	-12.90	22.32
Silver	2016	74	-12.84	22.41
Silver	2017	51	-12.50	15.11
Silver	2017	9	-12.85	18.59
Silver	2016	122	-12.62	17.85
Silver	2017	8	-13.14	21.66
Silver	2018	96	-13.26	23.69
Silver	2017	128	-13.16	23.91
Silver	2016	48	-13.80	18.67
Silver	2017	129	-12.10	19.42
Silver	2016	133	-12.96	18.04
Silver	2016	57	-12.61	20.29
Silver	2016	115	-13.40	20.55
Silver	2016	146	-12.14	20.66

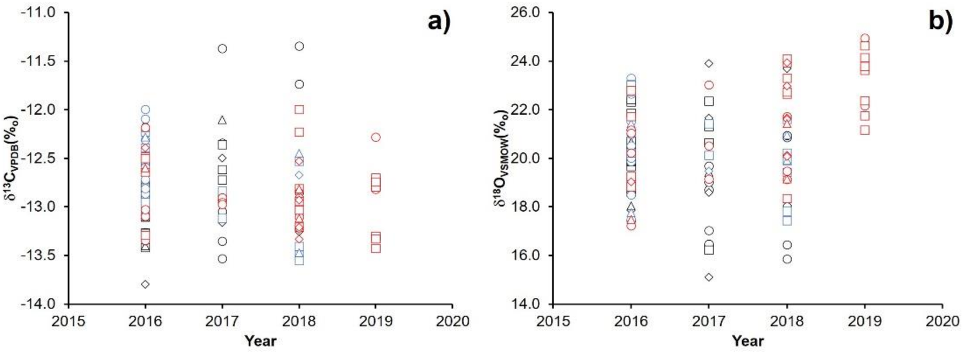

Figure 1. Graphical representation of the experimental data for a) $\delta^{13}C_{VPDB}$ and b) $\delta^{18}O_{VSMOW}$, according to the year of production of Tequila 100% agave silver (black), aged (blue), and extra-aged (red). Region: ○ Altos Sur, □ Valles, ◊ Cienega, Δ Centro.

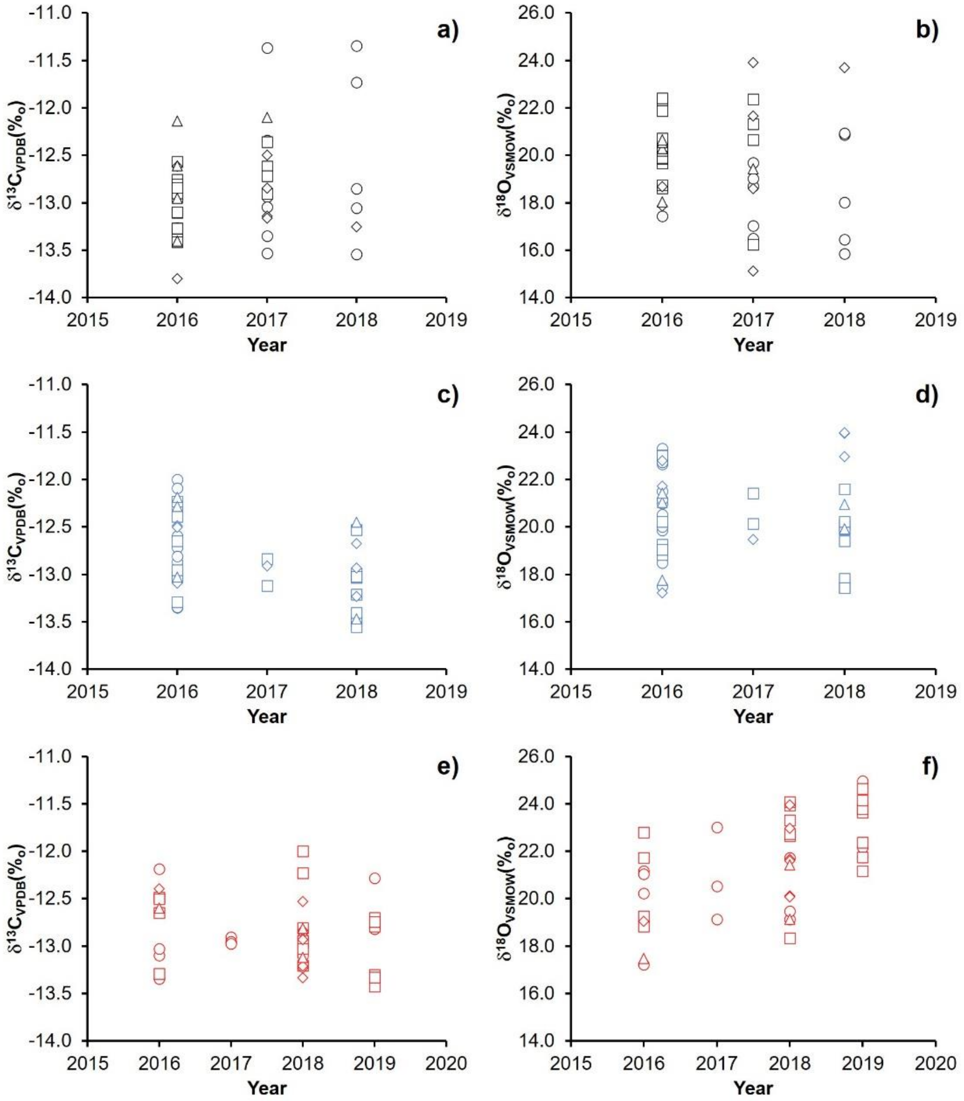

Figure 2. Graphical representation of the experimental data for $\delta^{13}C_{VPDB}$ and $\delta^{18}O_{VSMOW}$, according to the year of production of tequila 100% agave silver class (a and b), aged class (c and d), and extra-aged class (e and f). Region: ○ Altos Sur, □ Valles, ◊ Cienega, Δ Centro.
